# An update on the potential health benefits of carotenes 

**DOI:** 10.17179/excli2015-664

**Published:** 2016-01-06

**Authors:** Jae Kwang Kim

**Affiliations:** 1 Division of Life Sciences, College of Life Sciences and Bioengineering, Incheon National University, Incheon, 406-772, Korea

## Dear Editor,

Carotenes, which are yellow-orange pigments, are a class of related organic compounds classified as hydrocarbons, more specifically as terpenoids, with the molecular formula C40H56. Plants, fungi, and photosynthetic bacteria synthesize carotenes, while animals must obtain them as a dietary nutrient (Vrolijk et al., 2015[30]). Plants are capable of synthesizing several isomers of carotene. Alpha-carotene (α-carotene) and beta-carotene (β-carotene) are the two primary isomers found in plants; other carotene isomers found in plants are gamma-, delta-, epsilon-, and zeta-carotene (γ, δ, ε, and ζ-carotene) (Hammond and Renzi, 2013[9]). β-Carotene is the most common form of carotene in plants and can be found in yellow, orange, and green leafy vegetables and fruits. It is an important dietary resource and a precursor of vitamin A in humans (Haskell, 2012[12]; Tang, 2012[29]; Sommer and Vyas, 2012[28]).

Carotenes show a range of biological activity and health benefits for animals, making it an interesting material for the pharmaceutical, food, and cosmetics industries. We have reviewed the most recent studies on carotenes and its biological and pharmacological activities (Table 1(Tab. 1)) (References in Table 1: Li et al., 2015[17]; Freitas et al., 2015[7]; Sluijs et al., 2015[27]; Hashim et al., 2015[11]; Das et al., 2015[5]; Ben Amara et al., 2015[2]; Schnorr et al., 2014[25]; Fiorelli et al., 2014[6]; Lai et al., 2014[15]; Kasperczyk et al., 2014[13]; Lim et al., 2014[18]; Wang et al., 2014[31]; Kim et al., 2014[14]; Berti et al., 2014[3]; Gloria et al., 2014[8]; Orazizadeh et al., 2014[22]; Abdul-Hamid and Moustafa, 2014[1]; Nishida et al., 2014[21]; Harari et al., 2013[10]; Bjelakovic et al., 2013[4]; Lee et al., 2013[16]; Lin et al., 2013[20]; Rotenstreich et al., 2013[24]; Silva et al., 2013[26]; Lim et al., 2013[19]; Peng et al., 2013[23]). 

## Acknowledgements

This work was supported by the Incheon National University Research Grant in 2013.

## Figures and Tables

**Table 1:**
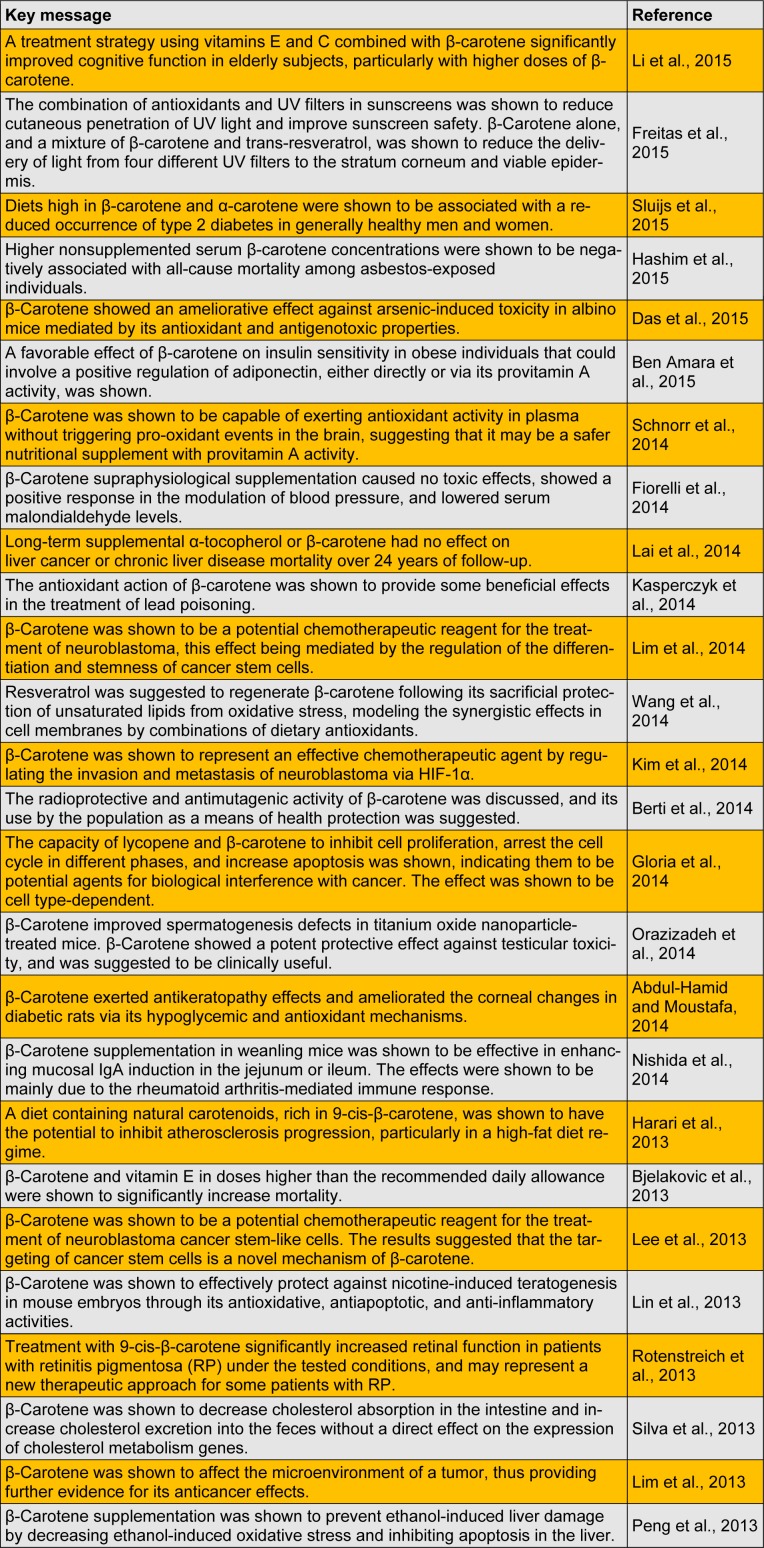
Recent studies on carotene and its biological and pharmacological activities
